# An ovine model for investigation of the microenvironment of the male mammary gland

**DOI:** 10.1111/joa.14055

**Published:** 2024-05-12

**Authors:** Benjamin P. Davies, Rachael C. Crew, Anna L. K. Cochrane, Katie Davies, André Figueiredo Baptista, Sonja Jeckel, Ian S. McCrone, Youguo Niu, Benjamin W. Strugnell, Katie Waine, Abigail L. Fowden, Clare E. Bryant, John W. Wills, Dino A. Giussani, Katherine Hughes

**Affiliations:** ^1^ Department of Veterinary Medicine University of Cambridge Cambridge UK; ^2^ Department of Physiology, Development and Neuroscience University of Cambridge Cambridge UK; ^3^ Department of Obstetrics and Gynaecology University of Cambridge Cambridge UK; ^4^ School of Human Sciences The University of Western Australia Perth Western Australia Australia; ^5^ Farm Animal Pathology and Diagnostics The Royal Veterinary College Hatfield UK; ^6^ Farm Post Mortems Ltd Durham UK; ^7^ Present address: Faculty of Veterinary Medicine University of Calgary Calgary Alberta Canada.

**Keywords:** male, mammary gland, microenvironment, model, sheep, udder

## Abstract

The specific biology of the male breast remains relatively unexplored in spite of the increasing global prevalence of male breast cancer. Delineation of the microenvironment of the male breast is restricted by the low availability of human samples and a lack of characterisation of appropriate animal models. Unlike the mouse, the male ovine gland persists postnatally. We suggest that the male ovine mammary gland constitutes a promising adjunctive model for the male breast. In this study, we evaluate the male ovine mammary gland microenvironment, comparing intact and neutered males. Assessment of the glandular histo‐anatomy highlights the resemblance of the male gland to that of neonatal female sheep and confirms the presence of rudimentary terminal duct lobular units. Irrespective of neutered status, cell proliferation in epithelial and stromal compartments is similarly low in males, and cell proliferation in epithelial cells and in the intralobular stroma is significantly lower than in pubertal female sheep. Between 42% and 72% of the luminal mammary epithelial cells in the male gland express the androgen receptor and expression is significantly reduced by neutering. Luminal epithelial cells within the intact and neutered male gland also express oestrogen receptor alpha, but minimal progesterone receptor expression is observed. The distribution of leukocytes within the ducts and stroma is similar to the mammary gland of female sheep and females of other species. Both macrophages and T lymphocytes are intercalated in the epithelial bilayer and are more abundant in the intralobular stroma than the interlobular stroma, suggesting that they may have a protective immunological function within the vestigial glandular tissue of the male sheep. Mast cells are also observed within the stroma. These cells cluster near the glandular tissue and are frequently located adjacent to blood vessels. The abundance of mast cells is significantly higher in intact males compared to neutered males, suggesting that hormone signalling may impact mast cell recruitment. In this study, we demonstrate the utility of the male ovine mammary gland as a model for furthering our knowledge of postnatal male mammary biology.

## Graphical abstract

The specific biology of the male breast remains relatively unexplored in spite of the increasing global prevalence of male breast cancer. In this study, we demonstrate the utility of the male ovine mammary gland as a model for furthering our knowledge of postnatal male mammary biology.

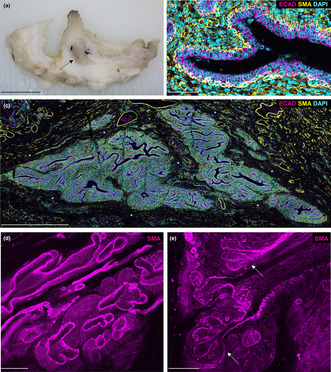

## 1 INTRODUCTION

The mammary gland nourishes and supports the development of offspring. Whilst they are intrinsically linked with female individuals, mammary glands persist in the majority of male mammals, although notable exceptions include mice, horses and marsupials (Cardiff et al., 2018; Hughes, 2021a; Renfree et al., 1990).

Early fetal development of the male mammary gland is typically consistent with that of the female, irrespective of species (Hassiotou & Geddes, 2013; Jenkinson, 2003; Pokharel et al., 2018). Between embryonic day 11 and 13 of fetal mouse development, mammary placodes are established from ectoderm thickening on bilateral milk lines (Macias & Hinck, 2012; Stewart et al., 2019). The mammary placodes then invaginate into the mesenchyme layer, forming mammary buds (Paine & Lewis, 2017). Sexual dimorphism of the mouse mammary gland is established at embryonic day 14 (Richert et al., 2000; Stewart et al., 2019). In male mice, androgen is produced from the developing testes which causes condensation of the mesenchyme within the developing mammary gland (Dürnberger & Kratochwil, 1980; Richert et al., 2000; Vandenberg et al., 2013). This results in the morphological distortion of the mammary epithelium detaching the gland from the overlying epidermis (Drews & Drews, 1977; Richert et al., 2000; Stewart et al., 2019). The gland thereafter regresses and at birth comprises minimal vestigial glandular tissue that lacks nipples (Cardiff et al., 2018; Pokharel et al., 2018; Szabo & Vandenberg, 2021). In some genetically modified mouse strains, glandular tissue can persist after birth (Pokharel et al., 2018; Szabo & Vandenberg, 2021) but may still lack key developmental structures required for mammary gland expansion such as terminal end buds (Kolla et al., 2017).

By contrast, human prepubescent breast development is consistent between the sexes until the influence of androgens at puberty limits both ductal and stromal expansion of male breast tissue (Hassiotou & Geddes, 2013; Jesinger, 2014). The male breast comprises a small arborising ductal tree, apparently largely without well‐developed lobules, embedded in an adipose‐rich stroma (Fox et al., 2022).

The male breast has the potential to develop similar pathologies to that of females but at a lower incidence (Chatterji et al., 2023; Iuanow et al., 2011). However, there has been less focus on the specific biology of the male breast. The prevalence of male breast cancer is increasing globally (Fox et al., 2022) and the risk of death is significantly higher than comparable female breast cancers (Liu et al., 2018). Male breast neoplasms also have different biology compared to breast neoplasms arising in women (Chatterji et al., 2023). The use of primate and non‐primate male mammary tumours as pre‐clinical models for male breast cancer has recently been discussed (Luo‐Yng Tay et al., 2024). However, research focussed specifically on the normal male breast is currently limited by both the low availability of human samples and the lack of characterisation of appropriate animal models in which the complete male mammary structure persists postnatally.

Expansion of the diversity of mammary gland models has been recently highlighted as an opportunity to develop our understanding of mammary functions and phenotypes that cannot be addressed using traditional models (Rauner, 2024). Sheep are commonly used to model human fetal development (Morrison et al., 2018) and we and others have noted anatomical similarities in mammary terminal duct lobular unit (TDLU) structure and stromal composition between the sheep mammary gland and the female breast (Hovey et al., 1999; Hughes, 2021b; Hughes & Watson, 2018; Nagy et al., 2021; Rowson et al., 2012). Male and female ovine fetuses exhibit parallel mammary development, with the gland and teat cistern present in both sexes around fetal day 80 (Jenkinson, 2003). However, there has been little examination of the male ovine mammary gland postnatally. We suggest that the mammary gland of the male sheep may constitute a promising model of the male human breast. Consequently, in this study, we evaluate the mammary microenvironment in the male sheep, comparing intact and neutered males to highlight how the male‐specific sex hormonal milieu may affect the mammary microenvironment.

## 2 MATERIALS AND METHODS

### 2.1 Animals

Ovine mammary tissue was obtained from both male and female sheep that were submitted to the diagnostic veterinary anatomic pathology services of either the Department of Veterinary Medicine, University of Cambridge or to the Royal Veterinary College. Mammary tissue was also collected during the post mortem examination of Welsh mountain sheep, euthanised for other research purposes under the Animals (Scientific Procedures) Act 1986. The Ethics and Welfare Committee of the Department of Veterinary Medicine, University of Cambridge, approved the study plan to use post mortem tissue in this project (references: CR223 and CR625). The nonregulated scientific use of post mortem mammary tissue collected from research animals was approved by the Named Veterinary Surgeon of the University of Cambridge. Any tissue containing evidence of mammary pathology was excluded from the study.

Male sheep used in this study ranged from 3 days old to 3 years old, but only animals older than 4 months were included in quantification analysis. Pubertal female sheep were aged from 4 to 11 months. Mature female sheep were all older than 2 years and were not expected to be oestrus cycling based on the time of year of the post mortem examination (sheep are seasonal breeders). The sheep used were from a variety of breeds (Table S1).

### 2.2 Tissue processing

Mammary tissue was fixed in 10% neutral‐buffered formalin for approximately 1 week. The entire male mammary gland and samples of female mammary parenchyma were then trimmed, processed using a routine histology protocol and embedded in paraffin. Sections were cut at 5 μm and mounted on coated glass slides (TOMO®) or stained with haematoxylin and eosin to check for microscopic pathology prior to inclusion in the study.

### 2.3 Dual immunohistochemistry and immunofluorescence

Formalin fixed paraffin‐embedded (FFPE) tissue was subjected to antigen retrieval using a PT link module and high pH antigen retrieval solution (both Dako Pathology/Agilent Technologies, Stockport, UK). For dual immunohistochemistry, an ImmPRESS® Duet Double Staining Polymer Kit (Vector Laboratories) was used. Primary antibodies were added at the appropriate concentration (Table S2) and incubated overnight at 4°C. Negative controls received isotype‐ and species‐matched immunoglobulins. Counterstaining was achieved by incubating in Mayer's Haematoxylin for 4 min. Slides were dehydrated in an ethanol and xylene series and Pertex® Mounting Medium was added dropwise. ClariTex Coverslips (24 × 50 mm) were then applied.

For immunofluorescence, slides were first incubated with 10% normal goat serum for 1 h at room temperature. Primary antibodies were added at the appropriate concentration (Table S2) and incubated overnight at 4°C. Slides were then incubated in darkness with secondary antibodies (Table S2) for 1 h at room temperature. Negative controls received isotype‐ and species‐matched immunoglobulins. Nuclei staining was performed by incubating with DAPI (10.9 μM) (SigmaAldrich/Merck Life Science UK Limited, Gillingham, UK) for 5 min. Slides were cover‐slipped using the Vectashield® VibranceTM Antifade mounting medium (catalogue H‐1700; Vector laboratories, Peterborough, UK) and imaged using a Leica TCS SP8 confocal microscope.

### 2.4 Clear, unobstructed brain imaging cocktails (CUBIC)

Mammary tissue was dissected and fixed in 10% neutral‐buffered formalin for 6–26 h. The gland was trimmed into smaller samples, approximately 15 × 15 × 5 mm. These samples were optically cleared using the CUBIC protocol (Lloyd‐Lewis et al., 2016; Susaki et al., 2014) with the modifications outlined below. Tissue was incubated in CUBIC reagent 1A for 4 days on a shaker at 37°C. The solution was replaced daily. Samples were then incubated overnight, on a shaker at 4°C, with a blocking solution containing normal goat serum [10% (volume per volume)] and Triton X‐100 [0.5% (weight per volume)] in phosphate‐buffered saline (PBS). Primary antibodies, diluted in blocking solution, were applied at appropriate concentrations (Table S2) and samples were incubated for 4 days on a shaker at 4°C. Tissue samples were subsequently thoroughly washed in PBS and secondary antibodies (Table S2), also diluted in blocking solution, were then applied. Samples were incubated in darkness on a shaker at 4°C. Negative controls received isotype‐ and species‐matched immunoglobulins. After further washing in PBS containing Triton X‐100 (0.1% [weight per weight]), nuclei staining was performed by incubating in DAPI (10.9 μM) (SigmaAldrich/Merck Life Science UK Limited, Gillingham, UK) at room temperature for at least 1 h. After further washing, the CUBIC reagent 2 was applied and incubated in darkness for 4 days on a shaker at 37°C. Tissue samples were imaged in Ibidi 35 mm glass bottom dishes (catalogue 81218‐200; ibidi GmbH, Gräfelfing, Germany) using a Leica TCS SP8 confocal microscope.

### 2.5 Slide scanning

Slides subjected to immunohistochemical staining were scanned at 40× magnification using a NanoZoomer 2.0RS, C10730, (Hamamatsu Photonics, Hamamatsu City, Japan). Scanned sections were analysed with viewing software (NDP.view2, Hamamatsu Photonics).

### 2.6 Sampling for cell proliferation and immune cell abundance

Slide scans were analysed with the NDP.view2 viewing software. Depending on the tissue area available for analysis, three to eight‐count boxes (400 × 230 μm) were randomly placed on each slide at 1.25× magnification (Figure S1). This size of count box was previously used to manually quantify proliferation within the ovine mammary gland (Nagy et al., 2021) and is large enough to sample approximately 5–10 mammary ductules. Boxes containing artefacts from slide cutting or scanning were repositioned.

All slide annotations and manual quantification detailed below were carried out by BPD and independently verified by an American board‐certified veterinary pathologist (KH).

### 2.7 Quantification of epithelial and stromal proliferation

Instances of myoepithelial and luminal cell proliferation, and proliferation of any cells located within the interlobular and intralobular stroma, as denoted by positive nuclear Ki67 staining, were manually counted (Figure S1b). The total count for all sample boxes on the slide was calculated. This total count was then normalised to the total sampled area (mm^2^) of glandular tissue, interlobular or intralobular stroma, calculated by tracing around the area using the NDP.view2 freehand annotation tool (Figure S1b). The protocol was repeated for all individuals within each experimental group and the mean was derived.

### 2.8 Manual quantification of hormone receptor expression

A Leica TCS SP8 confocal microscope was utilised to produce ×400 magnification tile scans of immunofluorescence slides. Tile scans were analysed using Fiji (Schindelin et al., 2012), and, depending on the size of the tissue, three to eight‐count boxes (each measuring 400 × 230 μm) were randomly placed on each slide at low magnification (Figure S2a). Hormone receptor positive luminal epithelial cell nuclei were manually counted and the total count for all sample boxes on the slide was calculated (Figure S2b). This count was expressed as a percentage of the total number of luminal epithelial cell nuclei within all sample count boxes. The protocol was repeated for all individuals within each experimental group and the mean percentage of hormone receptor positive luminal cell nuclei for each group was calculated.

### 2.9 Epithelial and stromal macrophage, T lymphocyte, and mast cell abundance

Using NDP.view2, the number of epithelial‐associated IBA1 positive‐macrophages and CD3‐positive T lymphocytes were counted (Figure S1c,d). Cells were considered epithelial‐associated if >50% of their cytoplasmic perimeter contacted the basement membrane. The total number of epithelial‐associated macrophages and T lymphocytes in all count boxes was calculated for each individual, together with the total number of epithelial cells, and the epithelial‐associated immune cell count was expressed per 100 luminal epithelial cells. Stromal macrophages, T lymphocytes and mast cells were considered to be interlobular and intralobular, respectively when >50% of their cytoplasmic perimeter was within the respective type of stroma (Figure S1c–e). The total number of macrophages, T lymphocytes and mast cells for each stroma type was calculated. This count was normalised to the total sampled area (mm^2^) of interlobular or intralobular stroma, determined using the NDP.view2 freehand annotation tool (Figure S1c–e). The protocol was repeated for all individuals within each experimental group and the mean was derived.

### 2.10 Statistical analysis

To assess statistical significance in comparisons between cell proliferation and comparisons between hormone receptor expression, a Kruskal–Wallis test was conducted. Statistical significance in the differences in immune cell abundance between intact and neutered males were assessed using a Mann–Whitney *U* test. To assess statistical significance in the differences in immune cell abundance between intra‐ and interlobular stroma, a Wilcoxon signed‐rank test was performed. All data were collected using Microsoft Excel and were analysed using R studio (RStudio: Integrated Development for R. RStudio, PBC, Boston, MA URL http://www.rstudio.com/). All data are presented as mean values + the standard deviation.

## 3 RESULTS AND DISCUSSION

### 3.1 The gross and histo‐anatomy of the male sheep mammary gland resembles that of the neonatal female

A total of 33 sheep were analysed in the study (Table S1); 19 of these sheep were male, comprising 8 intact males and 11 neutered males. These male sheep ranged between 3 days old to 3 years old, but only male sheep older than 4 months were included in analyses involving quantification. These male sheep were of several different breeds, including 4 Cheviot cross males, 3 Welsh mountain males, 3 Texel cross males and 1 of each of Jacob cross, New Zealand Romney, Suffolk, mule, mule cross and Shetland. For 3 male sheep, no breed information was available. Male sheep were compared to a group of 7 pubertal female sheep and 7 mature female ewes. Pubertal females ranged from 4 to 11 months in age. These ewes were of three different breeds comprising five Welsh mountain ewes and one each of Texel cross and Beltex. Mature females were all older than 2 years and all 7 were Welsh mountain ewes.

**FIGURE 1:**
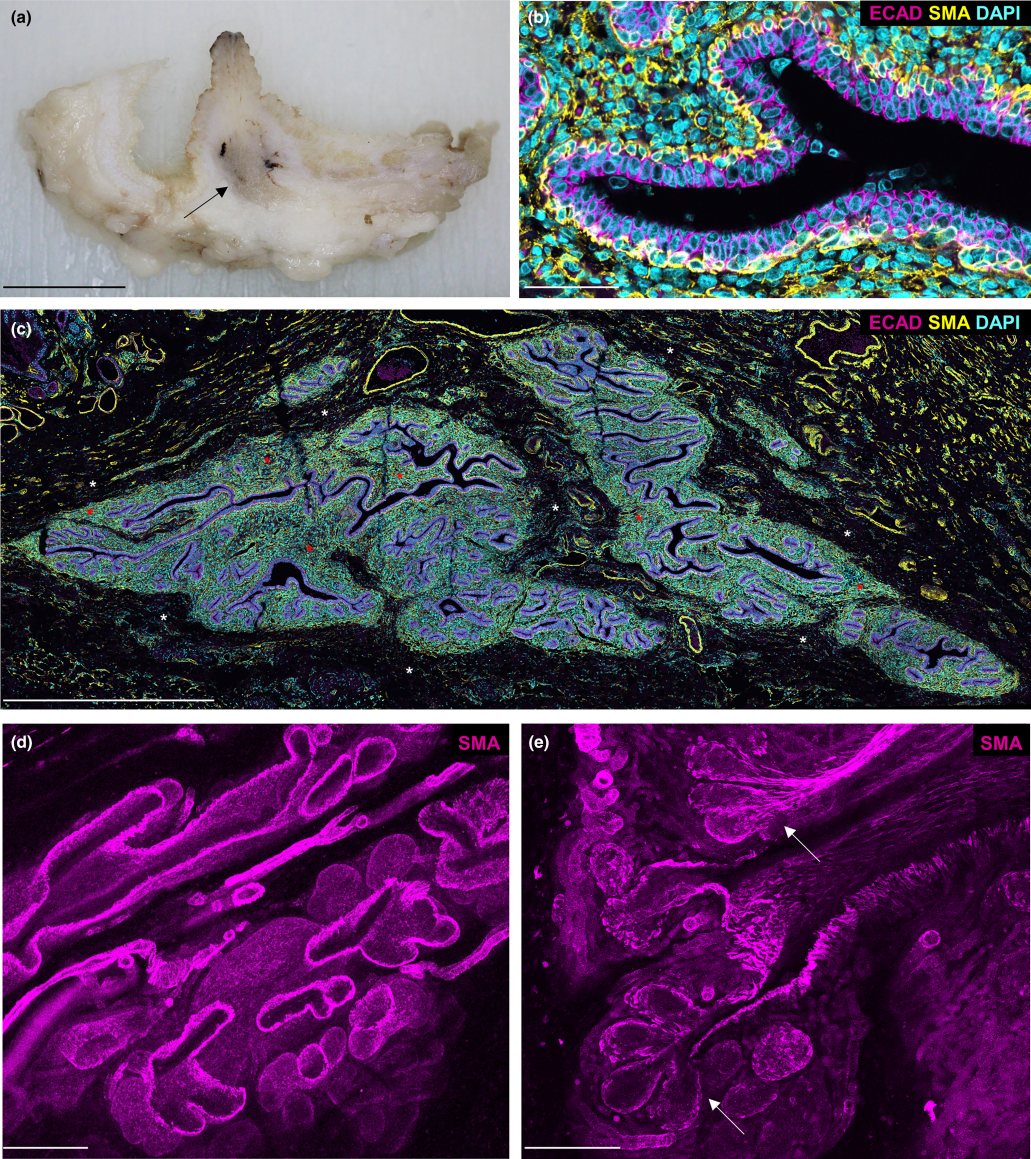
Macro and histo‐anatomy of the male ovine mammary gland. (a) A sub‐gross image of fixed male ovine mammary tissue. Arrow indicates mammary gland. (b, c) Immunofluorescence staining for luminal epithelial cells, using E‐cadherin (magenta), myoepithelial cells, using alpha‐smooth muscle actin (yellow) and DNA, using DAPI (cyan). Red asterisks indicate areas of intralobular mammary stroma. White asterisks indicate areas of interlobular mammary stroma. (d, e) 3D maximum intensity projections of optically cleared male ovine mammary tissue, using confocal microscopy. Immunofluorescence staining for alpha‐smooth muscle actin (magenta). Arrows indicate terminal duct lobular units. Images are representative of three biological repeats Scale bar = 1 cm (a); 50 μm (b); 1 mm (c); 200 μm (d); 100 μm (e).

To investigate the possibility that the mammary gland of the male sheep is a useful adjunct model for the male breast, we initially characterised the gross and histo‐anatomy of the gland. The male sheep mammary gland is located in the sub‐epithelial tissues below the teat (Figure 1a) and is composed of branching ducts that terminate in terminal duct lobular units (TDLUs) (Figure 1b–e). The walls of the ducts comprise a bilayer of luminal epithelial cells and basal myoepithelial cells (Figure 1b). Both intra‐ and inter‐lobular mammary stroma surround the ductal system (Figure 1c) and, like that of the female ruminant, express smooth muscle actin (Nagy et al., 2021; Safayi et al., 2012). Overall, the structure of the male gland is very similar to that of the neonatal female sheep (Hughes, 2021b; Nagy et al., 2021) and contrasts the minimal vestigial glandular tissue present in the postnatal male mouse mammary gland (Cardiff et al., 2018; Pokharel et al., 2018; Stewart et al., 2019; Szabo & Vandenberg, 2021).

### 3.2 The male ovine mammary gland exhibits minimal cell proliferation, irrespective of neutered status

**FIGURE 2:**
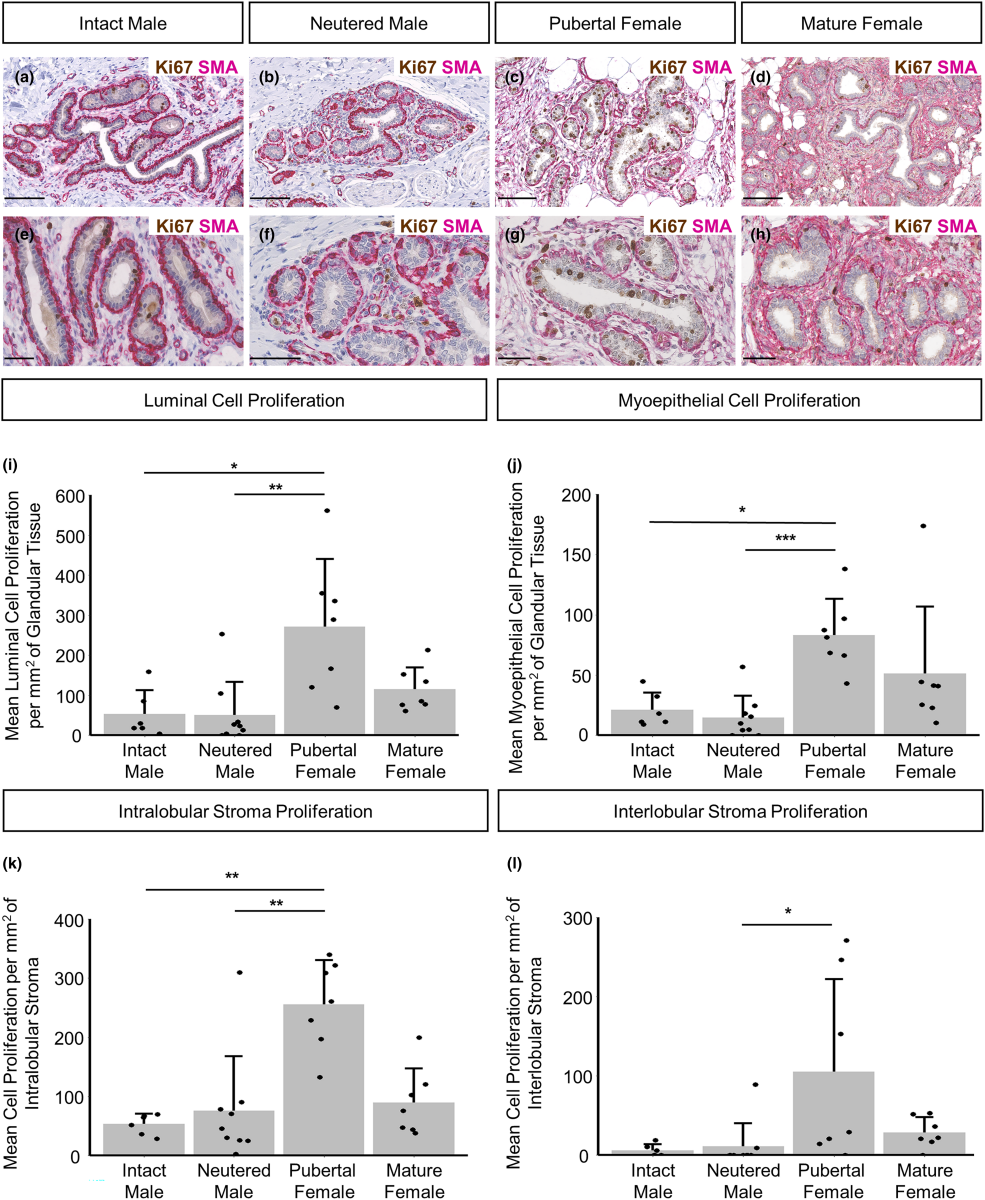
Proliferation dynamics within the intact male, neutered male, pubertal female and mature female mammary gland. (a–h) Dual immunohistochemical staining for Ki67 (brown) and alpha‐SMA (magenta) in the mammary gland of intact males (a, e), neutered males (b, f), pubertal females (c, g) and mature females (d, h). (i–l) Bar graphs illustrating differences in mean luminal cell proliferation (i), myoepithelial cell proliferation (j), intralobular stroma proliferation (k) and interlobular stroma proliferation (l) per mm^2^ of glandular tissue, intra‐ or interlobular mammary stroma + standard deviation (*p < 0.05, **p < 0.01, ***p < 0.001, *N* = 6 for intact males, *N* = 9 for neutered males, *N* = 7 for pubertal females, *N* = 7 for mature females, using Kruskal–Wallis test). Dots represent individual sheep. Images representative of 6 (a, e), 9 (b, f) and 7 (c, g, d, h) biological repeats. All IHC is shown with a haematoxylin counterstain. Scale bar = 100 μm (a–d); 50 μm (e–h).

Given that the female mammary gland undergoes dynamic changes in cell proliferation throughout its postnatal development cycle (Inman et al., 2015) we wished to assess the proliferation dynamics of different cellular compartments within the male gland, comparing intact and neutered male animals with pubertal females and mature females (Figure 2). Proliferation in both epithelial and stromal compartments is similarly low in male sheep irrespective of neuter status. Proliferation in luminal epithelial cells, myoepithelial cells and cells present in the intralobular stroma, is significantly higher in pubertal females when compared to both intact and neutered males (Figure 2i–l).

### 3.3 Neutering decreases mammary androgen receptor expression in sheep

**FIGURE 3:**
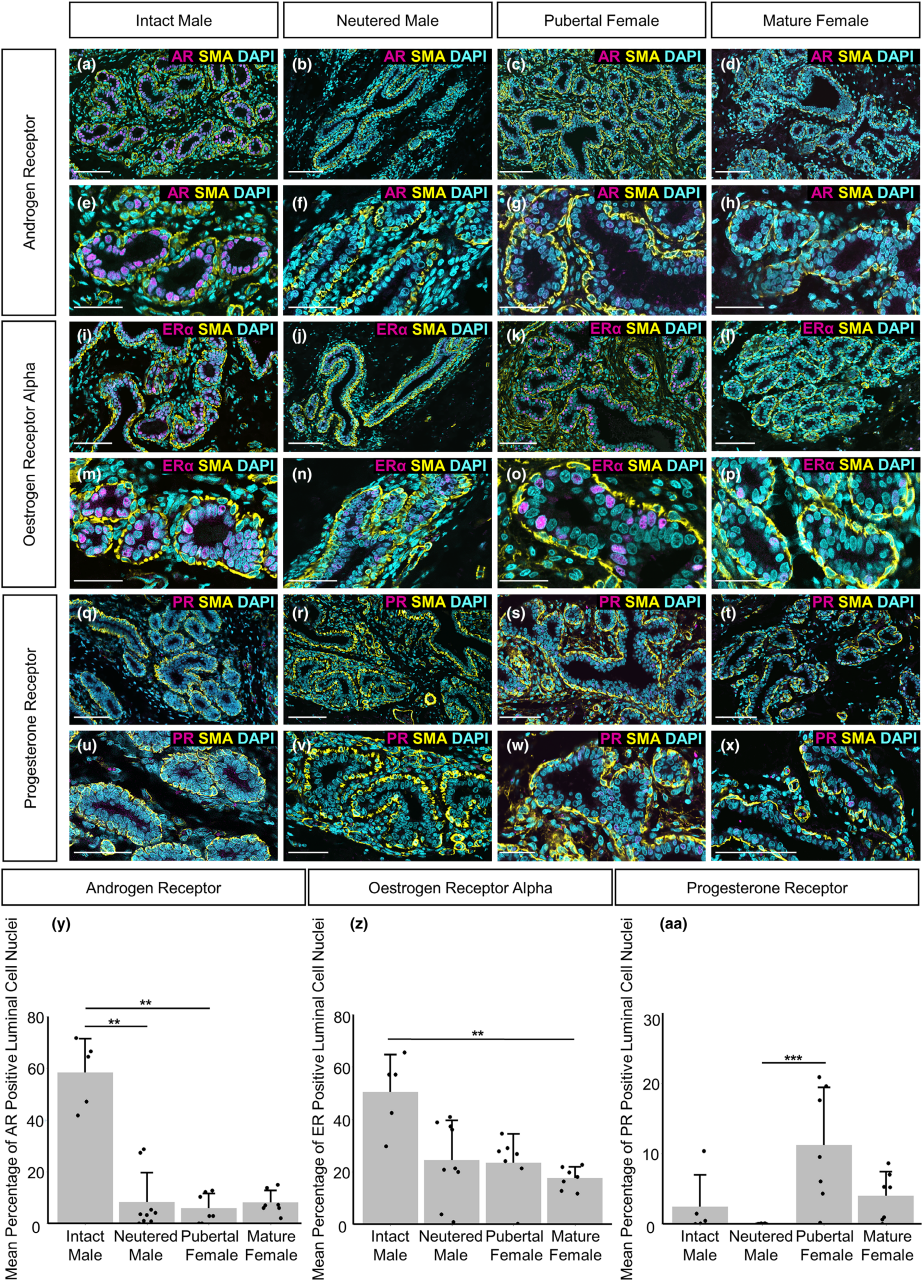
Differences in mammary epithelial hormone receptor expression between intact males, neutered males, pubertal females and mature females. (a–x) Immunofluorescence staining for androgen receptor (AR) (a–h), oestrogen receptor alpha (ERα) (i–p), progesterone receptor A/B (PR) (q–x) (magenta), alpha‐SMA (yellow) and DAPI (cyan) in the mammary gland of intact males (a, e, i, m, q, u), neutered males (b, f, j, n, r, v), pubertal females (c, g, k, o, s, w) and mature females (d, h, l, p, t, x). (y–aa) Bar graphs illustrating differences in the mean percentage of AR (y), ERα (z) or PR (aa) positive luminal cell nuclei + standard deviation (**p < 0.01, ***p < 0.001, *N* = 5 for intact males, *N* = 9 for neutered males, *N* = 7 for pubertal females, *N* = 7 for mature females, using Kruskal–Wallis test). Dots represent individual sheep. Images representative of a minimum of 5 biological repeats. Scale bar = 100 μm (a–d, i–l, q–t); 50 μm (e–h, m–p, u–x).

In our study population, between 42% and 72% of luminal mammary epithelial cells in intact male sheep express androgen receptor (AR) and expression is abrogated by neutering (Figure 3). Previous studies have illustrated that androgen receptor activation limits the expansion of glandular tissue within the female mammary gland. Transgenic mouse models in which the production or action of AR was ablated exhibited increased cell proliferation, ductal branching and number of terminal end buds (Gao et al., 2014; Simanainen et al., 2012). In humans, AR functions to limit the growth of breast tissue in both prepubescent boys and girls (Dimitrakakis & Bondy, 2009), and our data in the sheep are consistent with this observation.

Oestrogen receptor alpha (ER alpha) expression has previously been highlighted in epithelial cells within the TDLUs of prepubertal sheep (Colitti & Parillo, 2013) and is similarly observed in both male and female groups in our study (Figure 3i–p). Our analysis indicates that intact males have a higher percentage of luminal epithelial cells exhibiting ER alpha expression than mature females (Figure 3z), potentially reflecting a variable composition of luminal epithelial sub‐groups in the male gland compared to the female. Understanding the balance between AR and ER alpha expression in the male mammary gland is important because the vast majority of male breast cancer is ER‐positive (Cardoso et al., 2018; Chatterji et al., 2023).

Mean progesterone receptor expression is higher in pubertal females than neutered males (Figure 3aa). Progesterone receptor expression has been previously reported in the alveolar cells of the female ovine mammary gland during lactation (Colitti & Parillo, 2013) and prior research, using hormone‐treated murine mammary glands, indicates that progesterone receptor signalling promotes ductal branching during puberty (Atwood et al., 2000). Progesterone receptor signalling has also been shown to promote cell proliferation in multiple mammary cell types during puberty and pregnancy (Atwood et al., 2000; Brisken et al., 1998; Hilton et al., 2015). The low expression of progesterone receptor in neutered males and low but variable levels of progesterone receptor expression in intact males is consistent with the relatively limited tissue area occupied by the male gland and the lack of cell proliferation we observe within the male mammary gland (Figure 2i–l).

### 3.4 Macrophages and T lymphocytes are intercalated in the epithelial bilayer and cluster nearer ductal structures in the male ovine mammary gland

**FIGURE 4:**
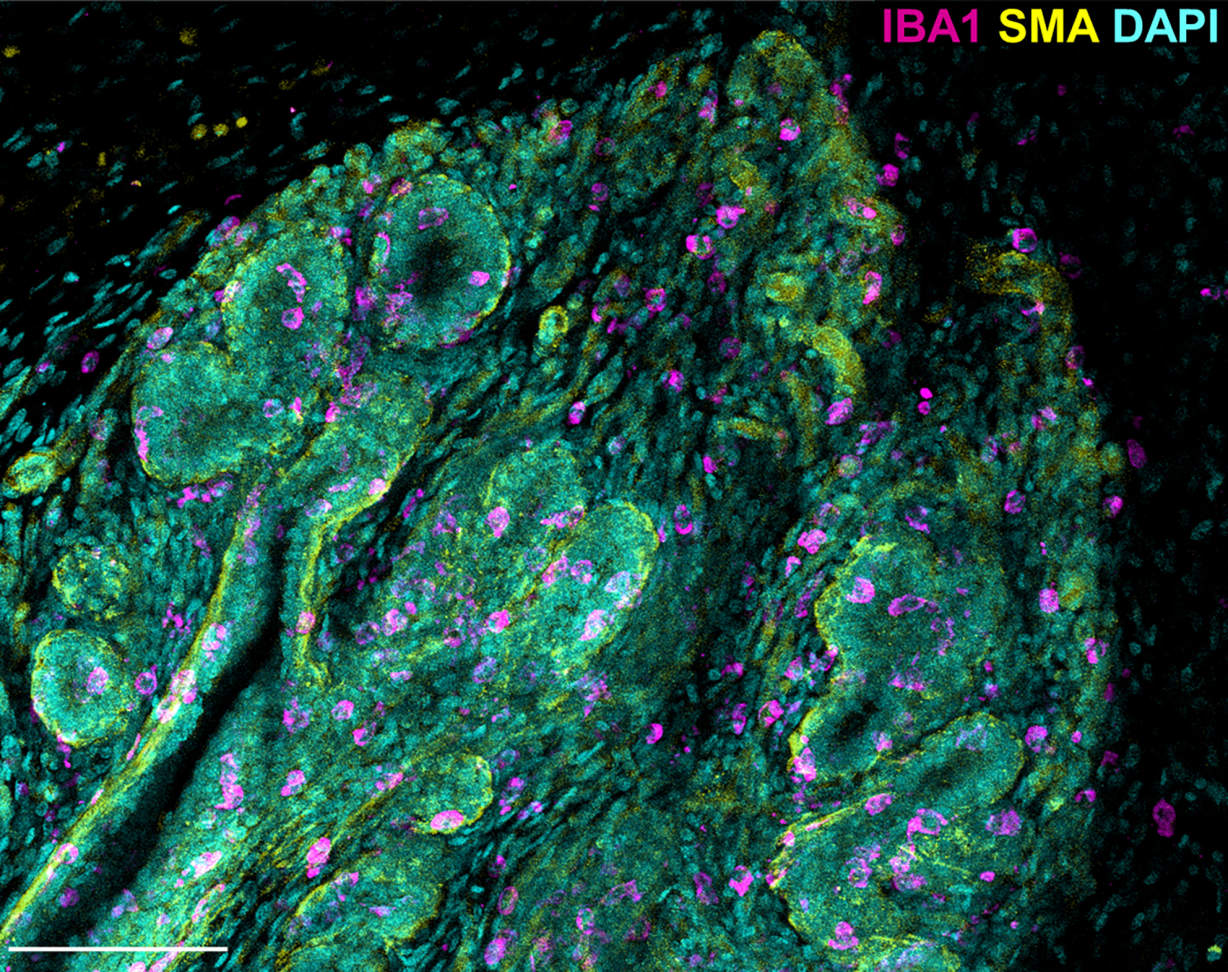
3D Macrophage distribution within the male ovine mammary gland. A 3D maximum intensity projection of optically cleared male ovine mammary tissue, using confocal microscopy. Immunofluorescence staining for IBA1 (magenta), alpha‐SMA (yellow) and DAPI (cyan). Image representative of 3 biological repeats. Scale bar = 100 μm.

**FIGURE 5:**
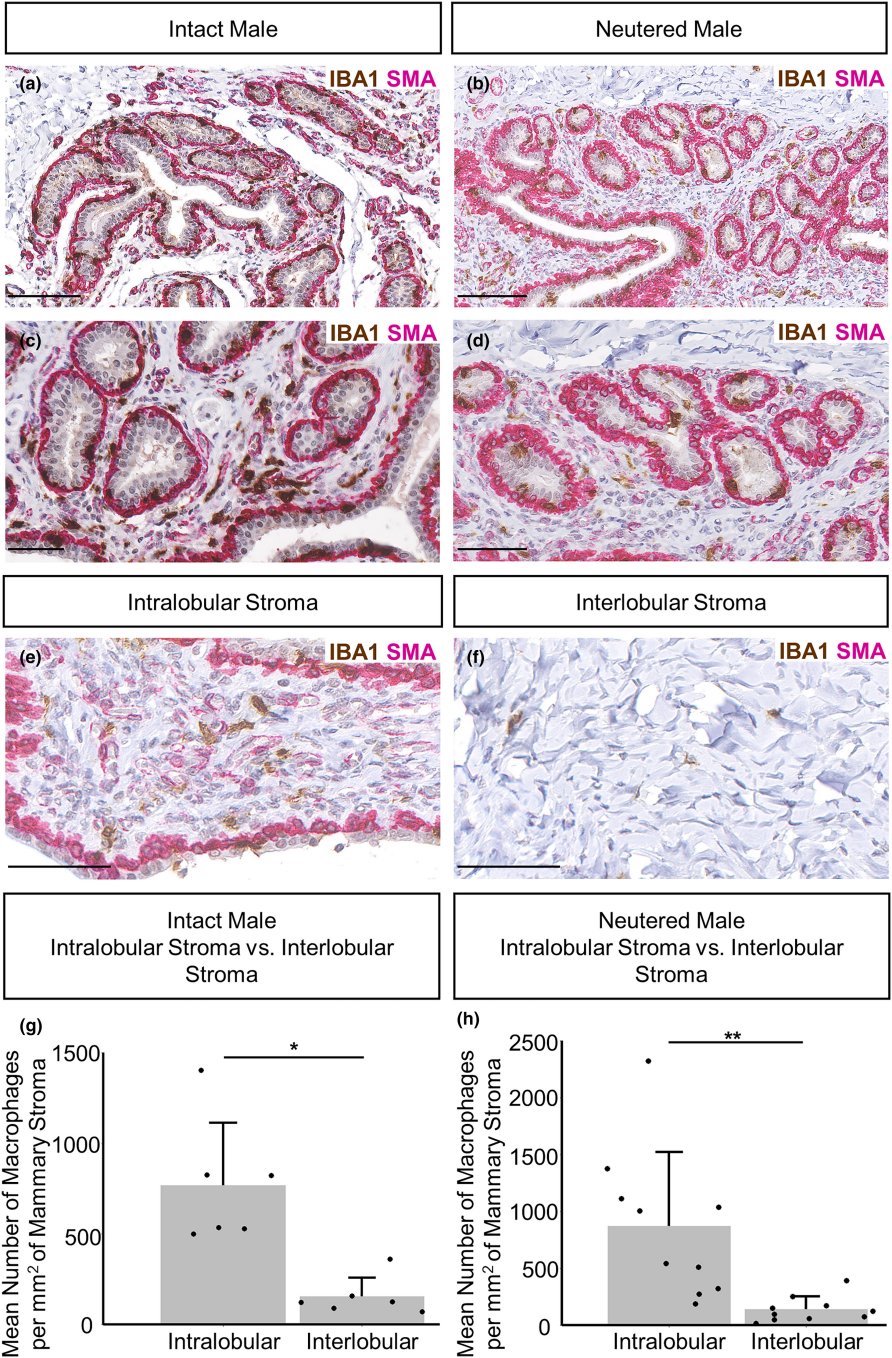
Macrophage abundance and localisation within the intact and neutered male ovine mammary gland. (a–f) Dual immunohistochemical staining for IBA1 (brown) and alpha‐SMA (magenta) in the mammary gland of intact males (a, c) and neutered males (b, d). (e–f) The abundance of macrophages in the intralobular (e) and interlobular mammary stroma (f). (g, h) Bar graphs illustrating differences in the mean number of macrophages per mm^2^ of mammary stroma in the intralobular and interlobular stroma of intact (g) and neutered males (h) + standard deviation (*p < 0.05, **p < 0.01, *N* = 6 for intact males, *N* = 10 for neutered males, using Wilcoxon signed‐rank test). Dots represent individual sheep. Images representative of 6 (a, c), 10 (b, d–f) biological repeats. Scale bar = 100 μm (a, b); 50 μm (c–f).

In both intact and neutered male sheep, IBA1 positive macrophages are intercalated in the epithelial bilayer and are present in the intra‐ and interlobular mammary stroma. There are abundant macrophages surrounding the TDLUs and many are intimately associated with the epithelium (Figures 4 and 5). The localisation of mammary macrophages in the male sheep is overall consistent with previous characterisation of the female neonatal and pubertal ovine mammary gland, with the exception that the macrophages within the male gland do not appear to exhibit the periodicity previously noted in females (Nagy et al., 2021). The intraepithelial macrophages of the male gland may be similar to the ductal macrophages reported in the mouse mammary gland (Dawson et al., 2020). These ductal macrophages have phenotypes and gene expression patterns distinct from stromal macrophages, reflecting an immune surveillance function (Dawson et al., 2020). The macrophages within the male ovine gland could have a similar role, but their precise phenotype and function requires further elucidation. In female mice, macrophage abundance also is affected by oestrogen and progesterone, during oestrus cycling (Chua et al., 2010; Hodson et al., 2013; Tower et al., 2022) and macrophages are receptive to androgen signalling (Liva & Voskuhl, 2001). However, our data suggest that male ovine mammary gland macrophage abundance is unaffected by neutered status (Figure S3), an observation warranting further future investigation.

In both intact and neutered males, the abundance of macrophages is significantly higher in the intralobular stroma, directly surrounding the glandular tissue, compared to the more distant interlobular stroma (Figure 5). This is consistent with previous examinations of the female bovine mammary gland (Beaudry et al., 2016).

**FIGURE 6:**
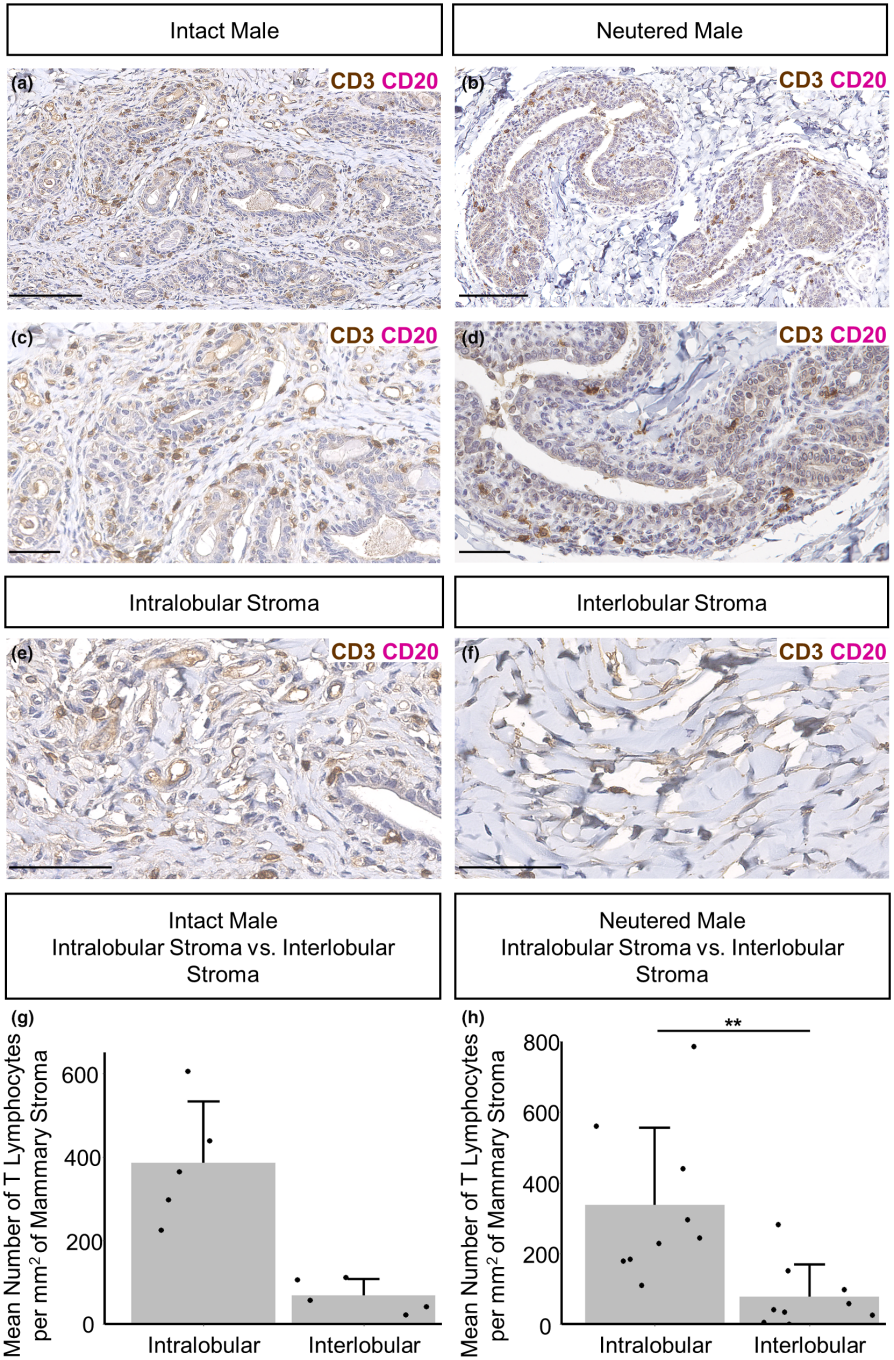
T‐lymphocyte abundance within the intact and neutered male ovine mammary gland. (a–f) Dual immunohistochemical staining for CD3 (brown) and CD20 (magenta) in the mammary gland of intact males (a, c) and neutered males (b, d) and within the intralobular (e) and interlobular mammary stroma (f). (g, h) Bar graphs illustrating differences in the mean number of T‐lymphocytes per mm^2^ of mammary stroma in the intralobular and interlobular stroma of intact (g) and neutered males (h) + standard deviation (**p < 0.01, *N* = 5 for intact males, *N* = 9 for neutered males, using Wilcoxon signed‐rank test). Dots represent individual sheep. Images representative of 5 (a, c, e, f) and 9 (b, d) biological repeats. All IHC is shown with a haematoxylin counterstain. Scale bar = 100 μm (a, b); 50 μm (c, f).

Similar to epithelial‐associated macrophages, CD3‐positive T lymphocytes are intercalated in the epithelial bilayer and are present in the intra‐ and interlobular mammary stroma. In contrast, there are few CD20‐positive B lymphocytes (Figure 6). Comparisons between intact and neutered males indicate that there is no significant difference in abundance of T lymphocytes associated with the epithelium or in the intra‐ or interlobular stroma (Figure S4). In neutered males, there are more T lymphocytes in the intralobular stroma than in the interlobular stroma (Figure 6). The clustering of both macrophages and T lymphocytes within and near the male mammary ductal structures may imply that they have an active immunological function within the male ovine vestigial glandular tissue, which has a potential portal for ingress of pathogens from the exterior through the teat.

### 3.5 Mast cells cluster nearer ductal structures in the male ovine mammary gland and their abundance is significantly higher in intact males

**FIGURE 7:**
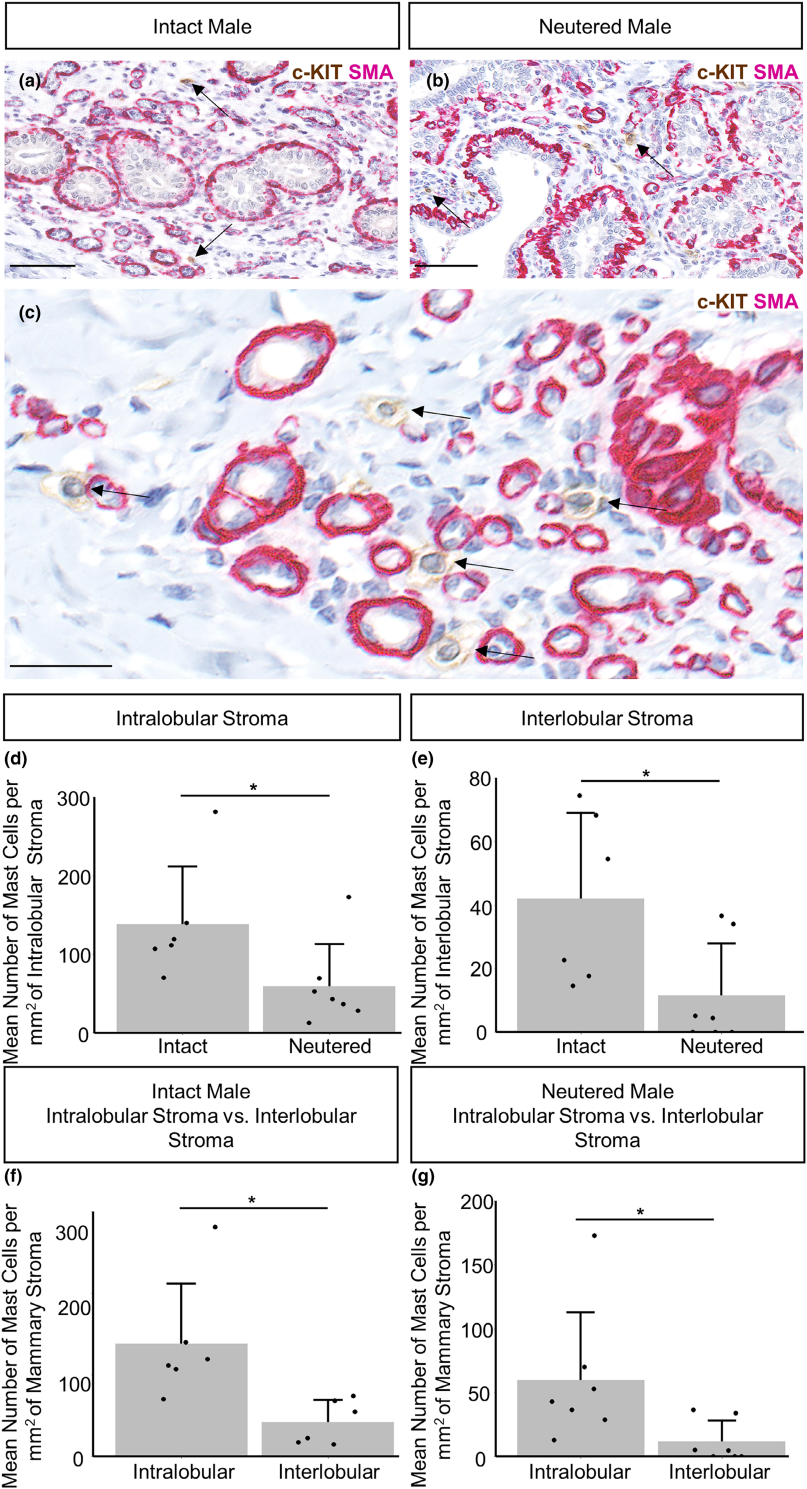
Mast cell abundance is significantly higher in intact males. (a–c) Dual immunohistochemical staining for c‐Kit (brown) and alpha‐SMA (magenta) in the mammary gland of intact males (a) and neutered males (b). Arrows indicate positive staining for mast cells. (c) Mast cells are located in close proximity to blood vessels in the male ovine mammary gland. Arrows indicate positive mast cell staining. (d, e) Bar graphs illustrating differences in the mean number of mast cells per mm^2^ of intralobular (d) and interlobular (e) stroma in intact and neutered males + standard deviation (*p < 0.05, *N* = 6 for intact males, *N* = 7 for neutered males, using Mann–Whitney *U* test). (f, g) Bar graphs illustrating differences in the mean number of mast cells per mm^2^ of mammary stroma in the intralobular and interlobular stroma of intact (f) and neutered males (g) + standard deviation (*p < 0.05, *N* = 6 for intact males, *N* = 7 for neutered males, using Wilcoxon signed‐rank test). Dots represent individual sheep. Images representative of 6 (a, c) and 7 (b) biological repeats. All IHC is shown with a haematoxylin counterstain. Scale bar = 50 μm (a, b); 25 μm (c).

Mast cells have been previously identified in the mammary gland of the mouse (Hughes et al., 2012; Lilla & Werb, 2010), rat (Ramirez et al., 2012) and cow (Beaudry et al., 2016). Typically, histological identification of mast cells is carried out using toluidine blue staining to highlight the cells' metachromatic staining granules. We and others have used toluidine blue to identify mast cells in the mammary gland of laboratory rodents (Hughes et al., 2012; Lilla & Werb, 2010; Ramirez et al., 2012). However, toluidine blue staining does not positively stain mast cells in the ovine mammary gland, potentially due to differences in granule composition (Figure S5). Consequently, we have stained for c‐Kit, a transmembrane tyrosine kinase receptor (Ribatti, 2018), that has been previously used to identify mast cells in human tissues (Lammie et al., 1994). Mast cells are present in the mammary stroma in both intact and neutered male glands and are present in close proximity to blood vessels (Figure 7). This localisation is consistent with previous descriptions of mast cells in the female mouse mammary gland (Lilla & Werb, 2010).

The abundance of mast cells in the intra‐ and interlobular stroma is significantly higher in intact males compared to neutered males (Figure 7d,e), suggesting that steroid hormone signalling may impact mast cell recruitment. A prior study highlighted that mast cells within the human skin express androgen receptors, but mast cell degranulation remained unchanged upon the administration of a testosterone treatment (Chen et al., 2010). However, the authors did not comment on how testosterone treatment may have affected mast cell abundance. There is also some evidence that oestrogen could affect mast cell recruitment in the female bovine mammary gland, where researchers identified a trend in which that mast cell number increases upon the exogenous oestrogen treatment (Beaudry et al., 2016). Elucidating how specific hormone receptor signalling may affect mast cell recruitment within the male ovine gland is a direction for further experimental investigation.

Consistent with the analysis of macrophage and T lymphocyte abundance, mast cell abundance is also significantly higher in the intralobular stroma, compared to the more distant interlobular stroma (Figure 7f,g). This is seen in both intact and neutered males. Similarly in the rat mammary gland mast cells tend to be located in the stroma surrounding the ducts (Ramirez et al., 2012). Mast cell number is also four times higher in the stroma adjacent to the mammary ducts, compared to more distant regions, in the female bovine mammary gland (Beaudry et al., 2016). This suggests that mammary mast cell localisation is similar between species and between males and females.

### 3.6 Conclusions

The ovine tissue analysed in this study was obtained from multiple sources, from sheep with different genetic backgrounds and maintained with different husbandry practices. Additionally, tissue obtained from pubertal females was collected from sheep euthanised throughout the year and their stage of the oestrus cycle at the time of tissue collection is unknown. Together, this constitutes a heterogeneous sample population which introduces considerable variability into the dataset. Arguably, this mirrors the heterogeneity in the mammary microenvironment in the human population.

Immunohistochemical and immunofluorescence staining constitute invaluable techniques to analyse protein localisation of large tissue sections, and have been widely applied in this study. However, spatial transcriptomics has been previously applied to the study of mammary biology (Twigger & Khaled, 2021) and its use would be valuable in the study of the male mammary microenvironment.

This study demonstrates the utility of the male ovine mammary gland as a tool to further our understanding of postnatal male mammary biology. This vestigial glandular structure contains a diverse set of immune cell types and exhibits distinct hormone receptor expression patterns, features that in both cases are affected by neutered status. Interestingly, the immune microenvironment of the male ovine gland appears to share features with that of the female gland in sheep and other species. This observation, together with the well‐documented histo‐anatomical similarities of the sheep mammary gland to the human breast (Hughes, 2021b; Nagy et al., 2021; Rowson et al., 2012) indicate that the male sheep mammary gland may be a useful adjunctive model of the male human breast. As mammary tumourigenesis is rare in sheep (Hughes, 2021b; Newman et al., 2021; Rauner, 2024), the male ovine gland does not offer a direct model for male breast cancer, but it will facilitate furthering understanding of normal male mammary biology to use in comparative studies. In addition, the relative resistance of the sheep to development of mammary tumours may be a further fruitful avenue of future comparative study (Hughes, 2023).

## AUTHOR CONTRIBUTIONS

BPD and KH contributed to the study concept and design. BPD, RCC, ALKC, KD, AFB, SJ, ISM, YN, BWS, KW, ALF, DAG and KH contributed to the acquisition of data. BPD, CEB, JWW, and KH contributed to data analysis and interpretation. BPD and KH drafted the manuscript. All authors contributed to critical revision of the manuscript and approved the final version.

## Supporting information


Figures S1–S5:
Tables S1 and S2.

## Data Availability

The data that support the findings of this study are available from the corresponding author upon reasonable request.
