# Supplementary material for: An ovine model for investigation of the microenvironment of the male mammary gland
Source: J Anat. 2024 May 12;245(3):405–19. doi: 10.1111/joa.14055 (PMC11306760; doi:10.1111/joa.14055)
Supplement: Supplementary file 1 — Figures S1–S5: Tables S1 and S2. [file JOA-245-405-s001.pdf]

An ovine model for investigation of the microenvironment of the male mammary gland

Supplementary Material

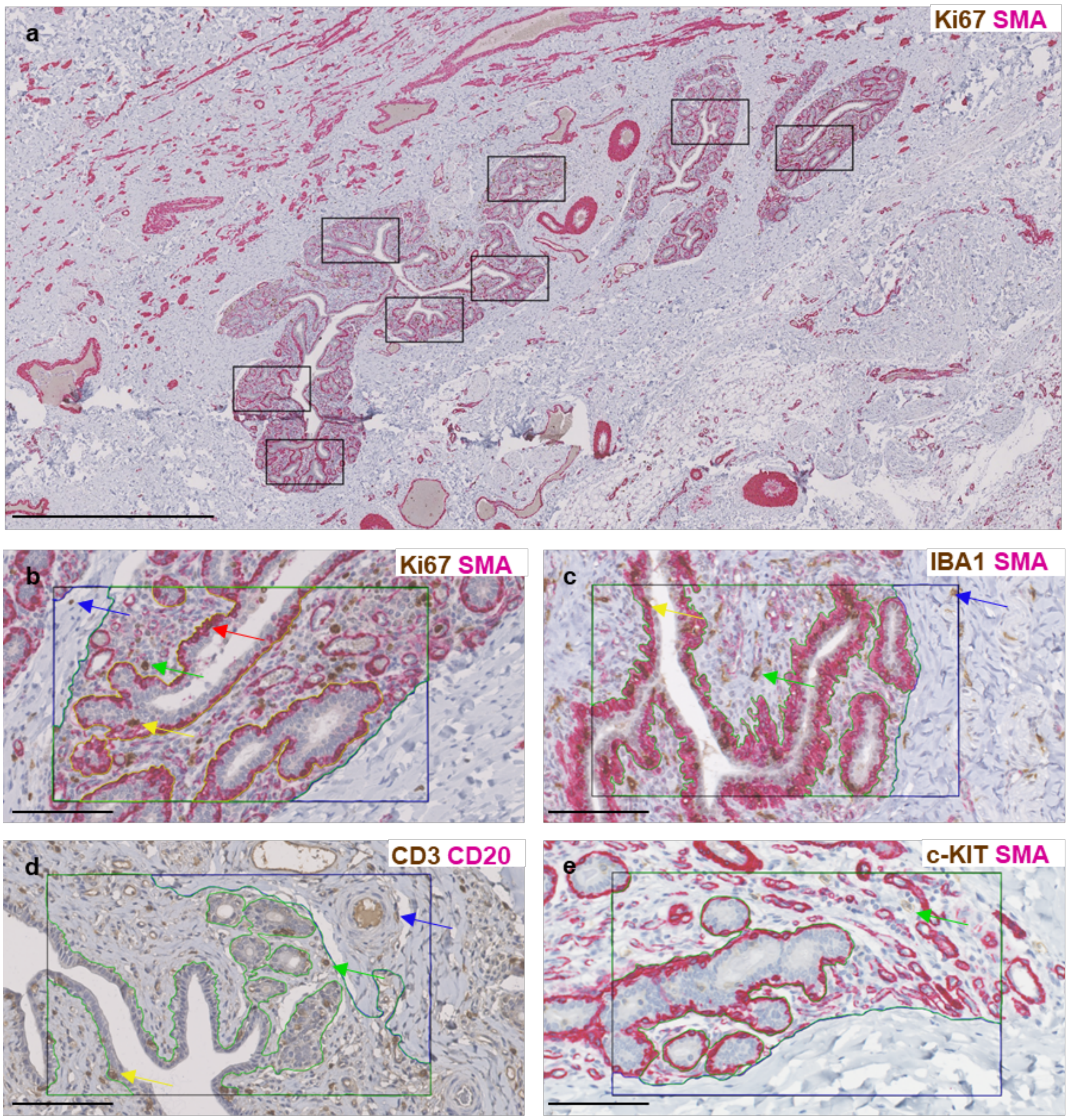

Supplementary Figure 1. Example sampling and quantification of cell proliferation and immune cell abundance within the ovine mammary gland. Dual immunohistochemical staining for Ki67 (brown) (a, b), IBA-1 (brown) (c), CD3 (brown) (d), c-Kit (brown) (e) and alpha-SMA (magenta) (a, b, c, e), CD20 (magenta) (d). (a) Eight count boxes (400 × 230 µm) randomly placed upon ovine mammary gland tissue, at 1.25x magnification. (b-e) Examples of count boxes (400 × 230 µm) at high magnification. Red arrow highlights instance of myoepithelial proliferation (b). Yellow arrows highlight instances of luminal proliferation (b), epithelial-associated IBA1 positive-macrophages (c) or epithelial-associated CD3-positive T lymphocytes (d). Green arrows highlight proliferation in the intralobular stroma (b), macrophages within the intralobular stroma (c), T lymphocytes within the intralobular stroma (d) or mast cells within the intralobular stroma (e). Blue arrows highlight proliferation in the interlobular stroma (b), macrophages within the interlobular stroma (c) or T lymphocytes

within the interlobular stroma (d). Yellow lines indicate glandular tissue area (mm^2) (b). Green lines indicate intralobular stroma area (mm^2) (b-e). Blue lines indicate interlobular stroma area (mm^2) (b-e). All calculated using the NDP.view2 freehand annotation tool. Images representative of 29 (a, b), 16 (c), 14 (d, e) biological repeats. All IHC shown with a haematoxylin counterstain. Scale bar = 1 mm (a); 100 µm (b-e).

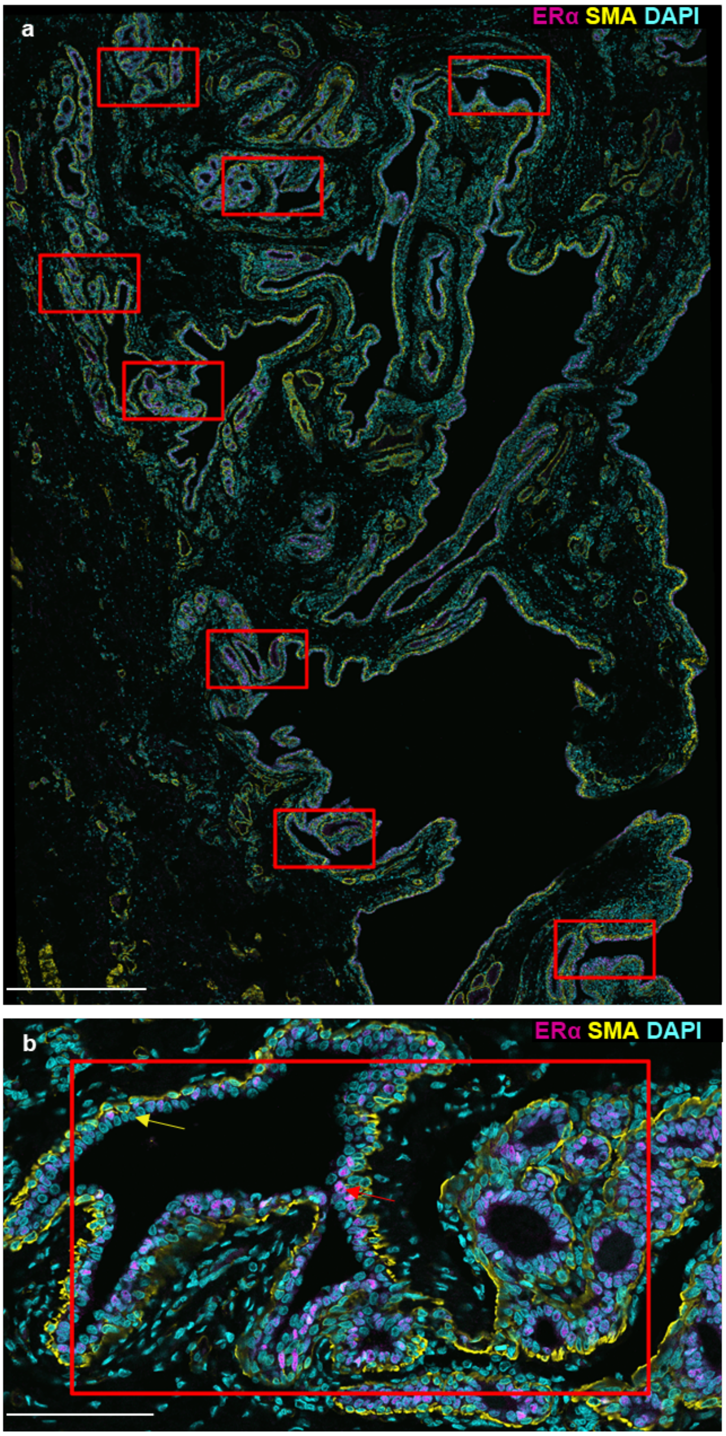

Supplementary Figure 2. Example sampling and quantification of hormone receptor expression within the ovine mammary gland. Immunofluorescence staining for oestrogen receptor alpha (ERα) (magenta) alpha-SMA (yellow) and DAPI (cyan) in the ovine mammary gland. (a) Eight count boxes (400 × 230 µm) are randomly placed. (b) An example of a count box (400 × 230 µm) at high magnification. Red arrow highlights ERα positive luminal cell. Yellow arrow highlights ERα negative luminal cell. Images representative of 28 biological repeats. Scale bar = 100 µm (a); 50 µm (b).

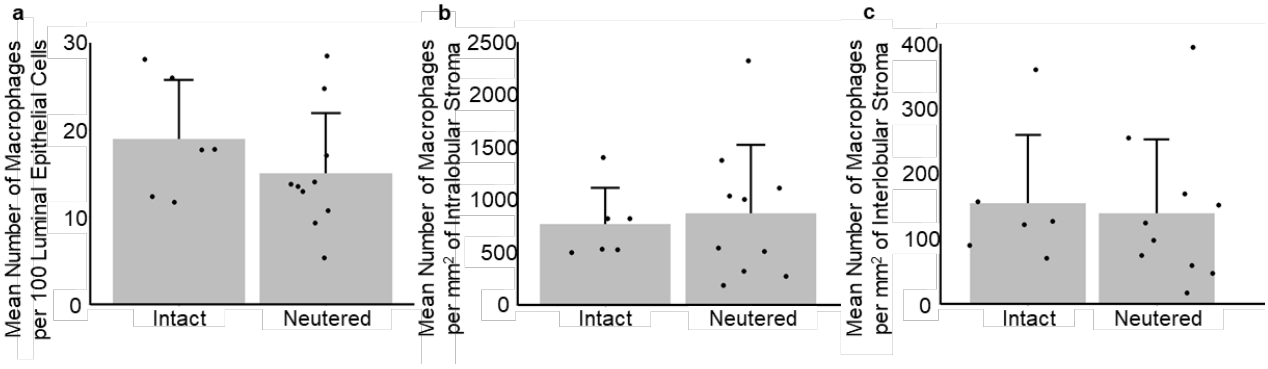

Supplementary Figure 3. There is no significant difference in macrophage abundance in the mammary glands of intact and neutered males. (a- c) Bar graphs illustrating differences, between intact and neutered males, in the mean number of macrophages per 100 luminal epithelial cells (a) and the mean number of macrophages per mm^2 of intralobular stroma (b) or interlobular stroma (c) + standard deviation. N= 6 for intact males, N =10 for neutered males. Mann-Whitney U test. Dots represent individual sheep.

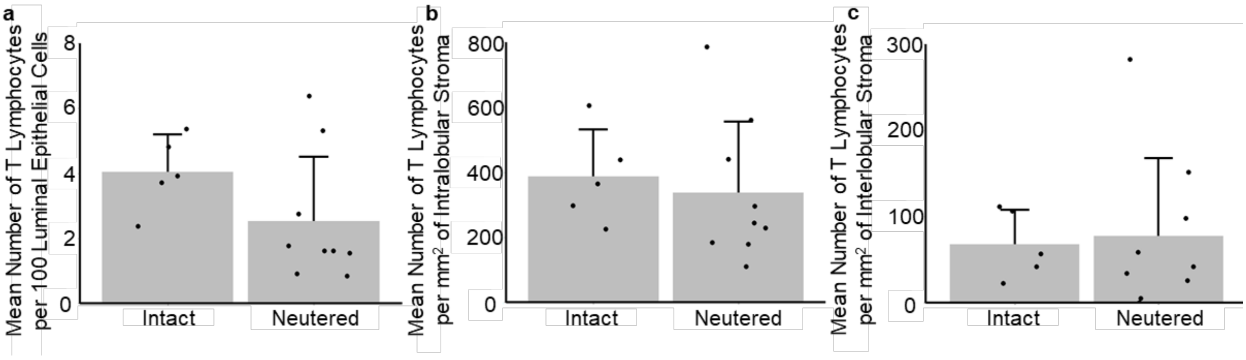

Supplementary Figure 4. There is no significant difference in T-lymphocyte abundance in the mammary glands of intact and neutered males. (a- c) Bar graphs illustrating differences, between intact and neutered males, in the mean number of T-lymphocytes per 100 luminal epithelial cells (a) and the mean number of T-lymphocytes per mm^2 of intralobular stroma (b) or interlobular stroma (c) + standard deviation. N= 5 for intact males, N =9 for neutered males. Mann-Whitney U test. Dots represent individual sheep.

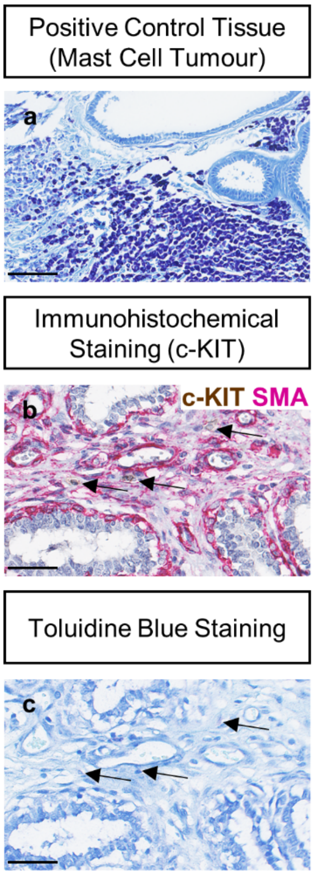

Supplementary Figure 5. Toluidine blue staining does not positively stain mast cells in the ovine mammary gland. (a) Toluidine blue staining in positive control tissue, a canine mast cell tumour. (b, c) Sequentially cut sections of FFPE ovine mammary gland tissue. (b) Dual immunohistochemical staining for c-Kit and alpha-SMA. Arrows indicate positive staining for mast cells. (c) Toluidine blue staining. Arrows indicate areas where positive toluidine staining should be. Images representative of 3 biological repeats. IHC shown with a haematoxylin counterstain. Scale bar = 100 µm (a); 50 µm (b, c).

Supplementary Table 1: Full details of sheep used in the study.

Sex	Source	Age	Breed	UK Meteorological Season Died/Euthanised	Neutered Status	Staining
Male	Diagnostic PM	7 weeks	Mule Cross	Spring	Intact	CUBIC (SMA), CUBIC (IBA1/SMA)
Male	Research	3 days	Welsh Mountain	Spring	Intact	CUBIC (SMA), CUBIC (IBA1/SMA)
Male	Research	Mature (2 years +)	Welsh Mountain	Winter	Intact	Ki67/SMA, AR/SMA, ER/SMA, PR/SMA, IBA1/SMA, CD3/CD20, cKit/SMA
Male	Diagnostic PM	6 months	Shetland	Summer	Neutered	Ki67/SMA, AR/SMA, ER/SMA, PR/SMA, IBA1/SMA, CD3/CD20, cKit/SMA
Male	Diagnostic PM	Mature (2 years +)	Unknown	Autumn	Intact	Ki67/SMA, AR/SMA, ER/SMA, PR/SMA, IBA1/SMA, CD3/CD20, cKit/SMA
Male	Diagnostic PM	9 months	Unknown	Winter	Neutered	IBA1/SMA
Male	Diagnostic PM	Mature (2 years +)	Unknown	Spring	Intact	Ki67/SMA, AR/SMA, ER/SMA, PR/SMA, IBA1/SMA, CD3/CD20, cKit/SMA
Male	Diagnostic PM	4 months	Mule	Summer	Neutered	Ki67/SMA, AR/SMA, ER/SMA, PR/SMA, IBA1/SMA, CD3/CD20, cKit/SMA
Male	Diagnostic PM	Mature (3 years)	Suffolk	Autumn	Intact	Ki67/SMA, IBA1/SMA, cKit/SMA
Male	Diagnostic PM	8 months	Cheviot Cross	Autumn	Neutered	Ki67/SMA, AR/SMA, ER/SMA, PR/SMA, IBA1/SMA, CD3/CD20, cKit/SMA
Male	Diagnostic PM	8 months	New Zealand Romney	Autumn	Neutered	Ki67/SMA, AR/SMA, ER/SMA, PR/SMA, IBA1/SMA, CD3/CD20, cKit/SMA
Male	Diagnostic PM	9 months	Cheviot Cross	Winter	Neutered	Ki67/SMA, AR/SMA, ER/SMA, PR/SMA, IBA1/SMA, CD3/CD20, cKit/SMA
Male	Diagnostic PM	10 months	Cheviot Cross	Winter	Neutered	Ki67/SMA, AR/SMA, ER/SMA, PR/SMA, IBA1/SMA, CD3/CD20, cKit/SMA
Male	Diagnostic PM	11 months	Cheviot Cross	Winter	Neutered	Ki67/SMA, AR/SMA, ER/SMA, PR/SMA, IBA1/SMA, CD3/CD20
Male	Diagnostic PM	14 months	Welsh Mountain	Spring	Intact	Ki67/SMA, AR/SMA, ER/SMA, PR/SMA, IBA1/SMA, CD3/CD20, cKit/SMA
Male	Diagnostic PM	8 months	Texel Cross	Winter	Neutered	Ki67/SMA, AR/SMA, ER/SMA, PR/SMA, IBA1/SMA, CD3/CD20, cKit/SMA
Male	Diagnostic PM	5 months	Texel Cross	Autumn	Intact	Ki67/SMA, AR/SMA, ER/SMA, PR/SMA, IBA1/SMA, CD3/CD20, cKit/SMA
Male	Diagnostic PM	7 weeks	Texel Cross	Spring	Neutered	CUBIC (SMA), CUBIC (IBA1/SMA)
Male	Diagnostic PM	Mature (2 years +)	Jacob Cross	Spring	Neutered	Ki67/SMA, AR/SMA, ER/SMA, PR/SMA, IBA1/SMA, CD3/CD20
Female	Diagnostic PM	5 months	Texel Cross	Summer	Intact	Ki67/SMA, AR/SMA, ER/SMA, PR/SMA
Female	Diagnostic PM	4 months	Beltex	Autumn	Intact	Ki67/SMA, AR/SMA, ER/SMA, PR/SMA
Female	Research	8 months	Welsh Mountain	Winter	Intact	Ki67/SMA, AR/SMA, ER/SMA, PR/SMA

Female	Research	11 months	Welsh Mountain	Winter	Intact	Ki67/SMA, AR/SMA, ER/SMA, PR/SMA
Female	Research	9 months	Welsh Mountain	Winter	Intact	Ki67/SMA, AR/SMA, ER/SMA, PR/SMA
Female	Research	9 months	Welsh Mountain	Spring	Intact	Ki67/SMA, AR/SMA, ER/SMA, PR/SMA
Female	Research	11 months	Welsh Mountain	Autumn	Intact	Ki67/SMA, AR/SMA, ER/SMA, PR/SMA
Female	Research	Mature (2 years +)	Welsh Mountain	Summer	Intact	Ki67/SMA, AR/SMA, ER/SMA, PR/SMA
Female	Research	Mature (2 years +)	Welsh Mountain	Summer	Intact	Ki67/SMA, AR/SMA, ER/SMA, PR/SMA
Female	Research	Mature (2 years +)	Welsh Mountain	Summer	Intact	Ki67/SMA, AR/SMA, ER/SMA, PR/SMA
Female	Research	Mature (2 years +)	Welsh Mountain	Summer	Intact	Ki67/SMA, AR/SMA, ER/SMA, PR/SMA
Female	Research	Mature (2 years +)	Welsh Mountain	Summer	Intact	Ki67/SMA, AR/SMA, ER/SMA, PR/SMA
Female	Research	Mature (2 years +)	Welsh Mountain	Summer	Intact	Ki67/SMA, AR/SMA, ER/SMA, PR/SMA
Female	Research	Mature (2 years +)	Welsh Mountain	Summer	Intact	Ki67/SMA, AR/SMA, ER/SMA, PR/SMA

Supplementary Table 2: Full details of primary and secondary antibodies utilised in immunohistochemical, immunofluorescence and CUBIC staining.

Target	Application (IHC, immunohistochemistry; IF, immunofluorescence; CUBIC, 3D tissue clearing)	Species and Clone (where stated)	Dilution	Manufacturer	Catalogue number
Primary antibodies					
E-cadherin	IF	Mouse monoclonal anti-human [NCH-38]	1:100	Dako/Agilent	M3612
Alpha Smooth muscle actin	Dual colour IHC, CUBIC	Mouse monoclonal anti-human [1A4]	1:500 (dual colour IHC), 1:100 (CUBIC)	Dako/Agilent	M0851
Alpha Smooth muscle actin	Dual colour IHC, IF	Rabbit monoclonal [EPR5368]	1:2000	Abcam	Ab124964
Ki67	Dual colour IHC	Mouse monoclonal anti-human [MIB-1]	1:100	Dako/Agilent	M7240
Androgen Receptor	IF	Rabbit monoclonal [EPR1535(2)]	1:100	Abcam	Ab133273
Oestrogen Receptor α	IF	Rabbit monoclonal [D6R2W]	1:200	Cell Signalling Technology	13258S
Progesterone Receptor A/B	IF	Rabbit monoclonal [D8Q2J]	1:1000	Cell Signalling Technology	8757S
IBA1	Dual colour IHC, CUBIC	Rabbit monoclonal [EPR16588]	1:1200 (dual colour IHC), 1:400 (CUBIC)	Abcam	Ab178846
CD3	Dual colour IHC	Mouse monoclonal [F7.2.38]	1:250	Dako/Agilent	M7254
CD20	Dual colour IHC	Rabbit monoclonal [E7B7T]	1:500	Cell Signalling Technology	48750S
c-Kit	Dual colour IHC	Rabbit monoclonal [D3W6Y]	1:100	Cell Signalling Technology	37805S
Secondary antibodies					
Mouse IgG, Alexa Fluor Plus 488	IF, CUBIC	Goat	1:500	Thermo Fisher Scientific	A32723
Rabbit IgG, Alexa Fluor Plus 647	IF, CUBIC	Goat	1:500	Thermo Fisher Scientific	A32733
